# Characterization of NO-producing neurons in the rat corpus callosum

**DOI:** 10.1002/brb3.218

**Published:** 2014-02-12

**Authors:** Paolo Barbaresi, Mara Fabri, Emanuela Mensà

**Affiliations:** Section of Neuroscience and Cell Biology, Department of Experimental and Clinical Medicine, Marche Polytechnic UniversityAncona, I-60020, Italy

**Keywords:** Colocalization, GFAP, immunocytochemistry, NADPH-d, nitric oxide, nNOS

## Abstract

**Introduction:**

The aim of this study was to determine the presence and distribution of nitric oxide (NO)-producing neurons in the rat corpus callosum (cc).

**Material and methods:**

To investigate this aspect of cc organization we used nicotinamide adenine dinucleotide phosphate diaphorase (NADPH-d) histochemistry and neuronal NO synthase (nNOS) immunocytochemistry.

**Results:**

Intense NADPH-d-positive (NADPH-d+) neurons were found along the rostrocaudal extension of the cc (sagittal sections). They were more numerous in the lateral cc and gradually decreased in the more medial regions, where they were very few or absent. The Golgi-like appearance of NADPH-d+ intracallosal neurons allowed dividing them into five morphological types: (1) bipolar; (2) fusiform**;** (3) round; (4) polygonal; and (5) pyramidal. The number of NADPH-d+ neurons (both hemispheres) was counted in two brains using 50-*μ*m thick sections. In the first brain, counts involved 145 sections and neurons were 2959; in the second, 2227 neurons were counted in 130 sections. The distribution and morphology of nNOS-immunopositive (nNOS_IP_) neurons was identical to that of NADPH-d+neurons. Some of these neurons were observed in the cc ependymal region, where they might be in contact with cerebrospinal fluid (CSF), monitoring its composition, pH, and osmolality changes, or playing a role in regulating the synthesis and release of several peptides. The somatic, dendritic, and axonal processes of many NADPH-d+/nNOS_IP_ neurons were closely associated with intracallosal blood vessels.

**Conclusions:**

Such close relationship raises the possibility that these neurons are a major source of NO during neural activity. As NO is a potent vasodilator, these findings strongly suggest that NO-positive neurons transduce neuronal signals into vascular responses in selected cc regions, thus giving rise to hemodynamic changes detectable by neuroimaging.

## Introduction

The earliest studies of the corpus callosum (cc), the largest neural pathway connecting the two cerebral hemisperes (Innocenti 1986), date back to 16th century. Considered for many centuries as the “seat of the soul” (Manzoni 1998), it took until the 18th century for Franz Joseph Gall and Johann Spurzheim to dissect alcohol-fixed brains and describe bundles of axons passing through the white matter and connecting the two hemispheres (Manzoni 2011).

The cc is made up of myelinated and unmyelinated axons and glial cells (Innocenti 1986). Callosal axons originate from pyramidal neurons located in layers II/III and V of the cerebral cortex (Innocenti 1986) and use glutamate as neurotransmitter (Barbaresi et al. 1987), released in the cc by unmyelinated fibers at specific axon–glia synaptic junctions (Ziskin et al. 2007). Moreover, an immunocytochemical and electrophysiological study provided evidence of physiologically active A1 adenosine receptors along callosal axons, likely responding to local adenosine release and influencing axonal transmission (Swanson et al. 1998).

Aside from glial cells, the cc also contains neurons. Studies of cc organization or reporting occasional data have described intracallosal neurons. Some multipolar neurons were described in the core and in the ventral part of human cc (Malobabic' et al. 1984) using the Golgi method; Riederer et al. (2004) and Revishchin et al. (2010) used immunocytochemical methods to study the localization of microtubule-associated protein 2 (MAP2) and calretinin-positive cells, respectively, in the cat and rat cc. A recent paper also described nitric oxide (NO)-producing neurons in the macaque cc (Rockland and Nayyar 2012).

Neuronal NO synthase (nNOS) is the enzyme responsible for NO synthesis from l-arginine (Vincent 1994) in central and peripheral nervous system neurons. A biochemical study showed that nicotinamide adenine dinucleotide phosphate diaforase (NADPH-d) and nNOS have the same molecular weight; both nNOS and NADPH-d activity was able to be immunoprecipitated from supernatants with NADPH-d-specific antiserum, and nNOS was competitively inhibited by the NADPH-d substrate, nitroblue tetrazolium (NBT). These data indicate that NADPH-d is a neuronal NOS (Hope et al. 1991).

For these reasons, we investigated the possible sites of NO synthesis in the rat cc using two different experimental approaches: NADPH-d histochemistry (NADPH-d_Hi_) and NOS immunocytochemistry (nNOS_Icc_). Therefore, the aim of this study was to describe the presence, distribution, and number of NO-producing neurons in the rat cc; moreover, as NADPH-_Hi_ gives Golgi-like images, we examined the morphology of such neurons. All data were then compared with those obtained in the monkey cc (Rockland and Nayyar 2012)**.**

There is evidence demonstrating NO production from nonneuronal cells in fibrous bundles similar to the cc. NADPH-d/NOS activity is found in glial cells in the optic nerve of normal guinea pig (Qi and Guy 1996). To gain insights into this aspect of cc organization, we performed fluorescent double-labeling experiments combining nNOS and glial fibrillary acidic protein (GFAP) immunocytochemistry.

Preliminary results have been presented to 63rd National Congress of the Italian Physiological Society (Mensà et al. 2012).

## Material and Methods

The study involved 21 adult male *Sprague-Dawley* albino rats (weight 250–300 g) whose care and handling was approved by the Animal Research Committee of Marche Polytechnic University in accordance with National Institutes of Health guidelines. All efforts were made to minimize animal suffering and to reduce the number of animals used.

### Light microscopy

#### NADPH-d histochemistry

Eleven animals (CC-NADPH-1/11) were deeply anesthetized with chloral hydrate (12% in phosphate buffer; PB, 0.1 mmol/L, pH 7.4) and then perfused through the left ventricle with saline followed by a mixture consisting of 2.5% glutaraldehyde and 0.6% paraformaldehyde (Takemura et al. 1996) in PB. After perfusion, brains were removed from the skull and placed in the same fixative for 12 h at 4°C. After postfixation, brains were cryoprotected in a sucrose solution (30% in 0.1 mmol/L PB at 4°C) until they sank and then freeze sectioned in the sagittal plane (three consecutive sections: one 60 *μ*m and two 40 *μ*m in thickness) on a sliding microtome (cases CC-NADPH-1/9). Sections for NADPHd-_Hi_ (60 *μ*m thick) were rinsed in PB (0.1 mmol/L; pH 8.0) and then transferred to a solution of 0.3% Triton X-100 in PB (0.1 mmol/L; pH 8.0) for 20–30 min. After this step, sections were processed for NADPHd-_Hi_ (Sigma Chemical Co, St. Louis, MO). They were incubated in PB containing 1 mg/mL NAPDH-d and 0.25 mg/mL NBT (Sigma Chemical Co, St. Loius, MO) for 1 h at 37°C in the dark, rinsed several times in PB, mounted on subbed slides, and air-dried; dehydrated in a graded series of alcohol and then coverslipped with DPX mountant. To establish cc boundaries, the first 40-*μ*m thick sections were reacted for cytochrome oxidase histochemistry (CO_HI_) and the second 40-*μ*m thick sections were mounted on subbed slides, air-dried and then counterstained with neutral red (1% in aqueous solution). CC-NADPH-d-10 and -11 were cut into 50-*μ*m thick sagittal sections; two sections (30 *μ*m thick) every 350 *μ*m were used for CO staining and neutral red counterstaining. Sections for NADPH-d_Hi_ were reacted as described above. Nomenclature and nuclear boundaries of the nervous tissue surrounding the cc were defined using the atlas of Paxinos and Watson (1982). Some sections were used for control experiments consisting of an incubation solution without NADPH-d or NBT; a positive reaction was never observed in these cases.

### Immunocytochemistry

#### nNOS experiments

Six rats (CC-nNOS-1/6) were transcardially perfused with saline followed by a solution of 4% paraformaldehyde, 0.5% glutaraldehyde, and 40% saturated picric acid in PB (0.1 mmol/L, pH 7.4). Brains were removed and postfixed for 12 h in the same fixative used for perfusion. After postfixation, brains were cryoprotected in increasing concentrations of a sucrose solution (10%, 20%, 30% in 0.1 mmol/L PB at 4°C) until they sank and then freeze sectioned in the sagittal plane (three consecutive sections, one 60 *μ*m and two 40 *μ*m in thickness) on a sliding microtome. Frozen sections 60 *μ*m in thickness from both hemispheres were used for nNOS_Icc_; the first 40-*μ*m thick sections were counterstained with neutral red (1% in aqueous solution); the second sections were reacted for CO_Hi_. Sections used for immunocytochemistry were first transferred to a solution of 3% H_2_O_2_ in phosphate-buffered saline (PBS) for 30 min to inhibit endogenous peroxidase activity, then incubated for 1 h in a blocking solution consisting of 20% normal goat serum (NGS) in PBS. After these steps, sections were rinsed several times in PB and then incubated overnight in primary antiserum (nNOS polyclonal antibody; 1:800; 3 h at room temperature and then overnight at 4°C). After washing in PB, sections were placed in a solution of biotinylated goat anti-rabbit (bGaR; diluted 1:100 in 1% NGS in PBS; Vector Laboratories, Burlingame, CA) for 1 h. Sections were washed again and then reacted with a solution containing avidin-biotin complex (diluted 1:100; Vector; Hsu et al. 1981). After several washes, sections were processed for peroxidase histochemistry using a 0.02% solution of 3,3′-diaminobenzidine tetrahydrochloride (DAB; Sigma) in 0.05 mmol/L Tris buffer, pH 7.6 (5 min). After a final rinse in PB, sections were mounted on subbed slides, dehydrated, and then coverslipped.

#### Immunofluorescence experiments

Two further animals (CC-Fl-1-2) were used for this series of experiments. Rats were deeply anesthetized with chloral hydrate and then transcardially perfused with saline followed by 4% paraformaldehyde in PB. After the brains were removed, they were postfixed overnight in the same fixative and then cut as described above into three consecutive sections (one 60 μm and two 40 μm thick). The former sections were first transferred to a solution of 3% H_2_O_2_ in PBS for 30 min, to inhibit endogenous peroxidase activity, and then incubated for 1 h in blocking solution. After these steps, sections were rinsed several times in PBS and then incubated overnight in a cocktail of primary antibodies containing GFAP made in mouse (1:1000) and nNOS made in rabbit (1:800). After washing in PB, sections were incubated in a mixture of species-specific secondary antibodies (1:150) conjugated to fluorescein (FITC) and rhodamine (TRITC; both from Invitrogen Chicago, IL) for 1 h at room temperature. Sections were washed in PB, mounted on slides, dried and coverslipped with Vectashield (Vector). Then 40*μ*m thick sections were reacted for CO_Hi_ and neutral red counterstaining. Control experiments were performed by omitting one or both primary and/or secondary antibodies. Sections were examined with an Eclipse-E600 microscope (Nikon Instech, Tokyo, Japan) equipped with a confocal imaging system (Microradiance, Bio-Rad, Hemel Hempstead, UK) provided with argon and helium/neon lasers (excitation 488 and 543 nm). Illustrations were prepared using Bio-Rad's LaserSharp image analysis program v. 3.2.

### Antibody characterization

The primary antibodies used in this study are listed in Table 1. The GFAP antibody (Clone GA5, MAB 360; Millipore, Billerica, MA) was made in mouse and raised against purified GFAP from porcine spinal cord; on western blot extracts from a human glioma cell line, it recognizes a band of about 51 kDa. The GFAP distribution in the cerebral and cerebellar cortex shown by the antibody was identical to a previous report (Taft et al. 2005).

**Table 1:** List of primary antibodies used in this study

Antigen	Immunogen	Manufacturer	Host-type	Dilution
GFAP clone GA5	GFAP isolated from porcine spinal cord	Millipore cat. no. MAB360	Mouse-monoclonal	1:1000
Neuronal NOS (nNOS)	Human nNOS amino acids 1422–1433	Cayman cat. no. 160870	Rabbit-polyclonal	1:800

The nNOS polyclonal antibody (160870; Cayman, Ann Arbor, MI) was made in rabbit against a peptide corresponding to amino acids 1422–1433 of human nNOS, and has successfully been used in a previous study. On western blots of protein extracts from rat mesencephalon, it reacted with a band of 155–160 kDa (Barbaresi et al. 2012) as specified by the manufacturer (see the technical information data sheet).

### Cytochrome oxidase (CO) staining

Two rats were used to verify the possibility of using the technique in this series of experiments. Animals were perfused and tissues postfixed and cut as described above. Brains were freeze sectioned in the sagittal plane. Two consecutive sections, one per stereotaxic plane, were used in two different ways. The first sections (60 *μ*m thick) were incubated at 37°C in the dark for 10–12 h in a solution containing 50 mg DAB, 30 mg cytochrome C (Type III, Sigma), and 4 g sucrose dissolved in 90 mL PB (0.1 mmol/L, pH 7.4; Wong-Riley 1979). Incubation was arrested when a clear differentiation between cerebral cortex and cc was visible. Sections were rinsed many times in PB, mounted on subbed slides, air-dried, dehydrated in xylene and then coverslipped. The adjacent sections (40 *μ*m thick) were counterstained with neutral red (1% in aqueous solution) and then coverslipped. Selected sections from CC-NADPH-1/11, CC-nNOS-1/6 and CC-Fl-1 and -2 were used for CO staining as described above.

### Data analysis

The distribution of NADPH-d+ and NOS_IP_ neurons in the cc was obtained using a camera lucida attached to a Leitz Orthoplan microscope equipped with a 25× objective. Callosal boundaries were obtained by comparing the sections reacted for CO_Hi_ with those counterstained with neutral red. The reconstructions thus obtained were then compared with those of the atlas of Paxinos and Watson (1982). Counts were performed in serial sagittal sections from two animals (CC-NADPH-10-11; see Table 2). The total number of intracallosal NADPH-d+ neurons was calculated on sections from both hemispheres starting from the lateral 4.5 sagittal plane of one hemisphere to reach the same stereotaxic plane in the contralateral hemisphere.

**Table 2:** (A) Arbitrary subdivisions of the rat cc, (B) Number of NADPH-d+ neurons in the three subdivisions of the rat cc

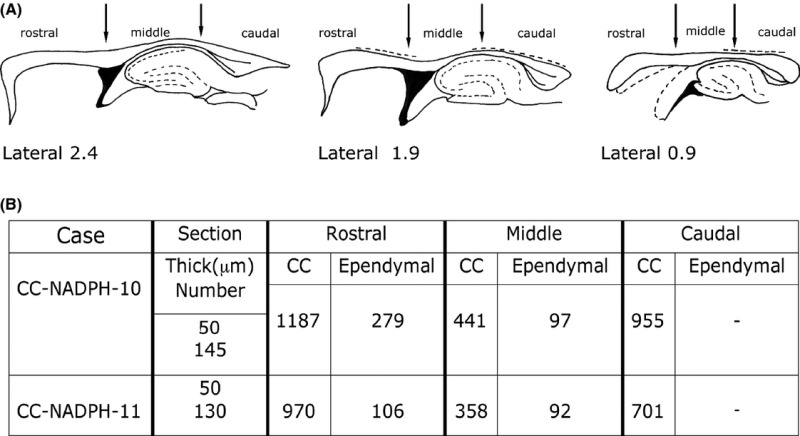

Microscopic studies of the morphology and percentage of intracallosal neurons (three cases: CC-NADPH-d-5; -7; -9; see Tables 3, 4) were performed using staining criteria similar to those of previous Golgi and NADPH-d studies (Jacobs and Scheibel 1993; Jacobs et al. 1993; Phillips et al. 2000; Barrera et al. 2001). Selected neurons, drawn using a camera lucida and a 100× oil immersion objective, exhibited the following characteristics: (1) labeled neurons had a clearly distinguishable morphology; (2) cell bodies had a central location within the 60-*μ*m section depth to minimize the cutting of dendritic branches near the section surface; (3) dendrites were not overly obscured by other heavily stained processes from nearby cells; (4) dendritic trees were intensely labeled and did not show discontinuity with their cell bodies. Using these criteria, we counted the percentage of each morphological type of intracallosal NADPH-d+ neurons, of each morphological type of intracallosal NADPH-d+ neurons close to blood vessels, and of each morphological type of intracallosal NADPH-d+ neurons crossing the cc toward the overlying cerebral cortex.

**Table 3:** Number and percentage of subtypes of intracallosal NADPH-d+ neurons

	Bipolar				Pyramidal		
					
Case	Fusiform *N* (%)	Rectangular *N* (%)	Round *N* (%)	Polygonal *N* (%)	Triangular *N* (%)	Pyriform *N* (%)	Total
CC-NADPH-5	105 (22.38)	17 (3.62)	106 (22.60)	142 (30.27)	37 (7.88)	62 (13.21)	469
	122 (26.01)				99 (21.10)		
CC-NADPH-7	147 (25.00)	26 (4.42)	106 (18.02)	176 (29.93)	65 (11.05)	68 (11.56)	588
	173 (29.42)				133 (22.61)		
CC-NADPH-9	102(26.08)	9 (2.30)	67 (17.13)	118 (30.17)	53 (13.55)	42 (10.74)	391
	111 (28.38)				95 (24.29)		
Total	354 (24.44)	52 (3.59)	279 (19.26)	436 (30.11)	155 (10.70)	172 (11.87)	1448
	406 (28.03)				327 (22.58)		

**Table 4:** Number and percentage of subtypes of intracallosal NADPH-d+ neurons close to blood vessels

	Bipolar				Pyramidal		
					
Case	Fusiform *N* (%)	Rectangular *N* (%)	Round *N* (%)	Polygonal *N* (%)	Triangular *N* (%)	Pyriform *N* (%)	Total (%)/Total NADPH
CC-NADPH-5	38 (22.89)	4 (2.40)	32 (19.27)	62 (37.34)	15 (9.03)	15 (9.03)	166 (35.39)/469
	42 (25.30)				30 (18.07)		
CC-NADPH-7	60 (25.86)	8 (3.44)	66 (28.44)	58 (25.00)	27 (11.63)	13 (5.60)	232 (39.45)/588
	68 (29.31)				40 (17.24)		
CC-NADPH-9	45 (28.84)	1 (0.64)	33 (21.15)	43 (27.54)	16 (10.25)	18 (11.53)	156 (39.89)/391
	46 (29.48)				34 (21.79)		
Total	143 (25.81)	13 (2.34)	131 (23.64)	163 (29.42)	58 (10.46)	46 (8.30)	554 (38.25)/1448
	156 (28.15)				104 (18.77)		

## Results

### Distribution of NO-producing neurons

To improve the visualization of cc boundaries, avoiding confusion with the overlying noncallosal white matter, the two sections adjacent to those reacted for NADPH_Hi_ or NOS_Icc_ were processed for CO activity and counterstained with neutral red.

**Figure 1:**
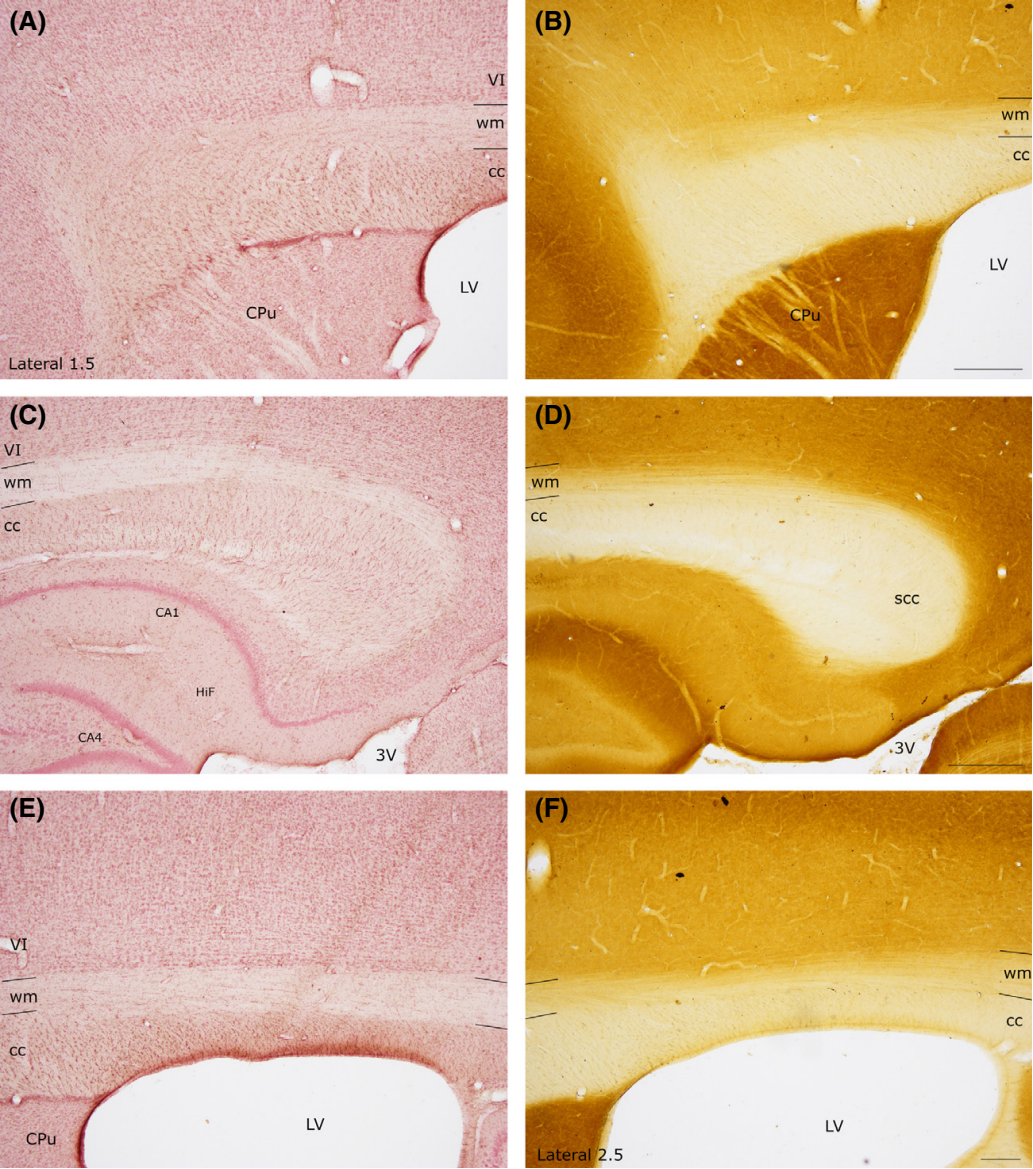
Comparison of cc borders defined by CO activity and those defined by neutral red counterstaining. (A–C) Anterior and posterior part of the rat cc (same mediolateral level). Neutral red counterstaining. (B–D) CO reaction, section adjacent to the one shown in (A–C). (E–F) Central part of the rat-cc. (E) Neutral red; (F), CO staining. Adjacent sections. Stereotaxic coordinates according to the atlas of Paxinos and Watson (1982) on bottom left side. VI, sixth layer of the cerebral cortex; wm, white matter. Calibration bars: 500 *μ*m.

Different levels of CO activity were observed that peaked in the cerebral cortex with a dark brown reaction; its intensity diminished in the direction of the underlying white matter, where CO activity was very low (Fig. 1). As CO activity was even lower in the cc, comparison of sections stained for CO_HI_ with adjacent sections counterstained with neutral red made it possible to define and reconstruct, with the help of the camera lucida, the cc boundaries and to compare them with those of the atlas Paxinos and Watson (1982).

In all rat brains, numerous cc neurons were positive for NADPH-d_Hi_ or NOS_Icc_. They were abundant along the rostrocaudal dimension of the cc but showed regional variations along its lateromedial extension. As shown in Figures 3 and Figure 4A, NADPH-d+/NOS_IP_ neurons were numerous in the lateral regions and progressively diminished in the medial cc, where they were very few or absent (Figs. 3 and Fig. 4B–B′, D).

**Figure 2:**
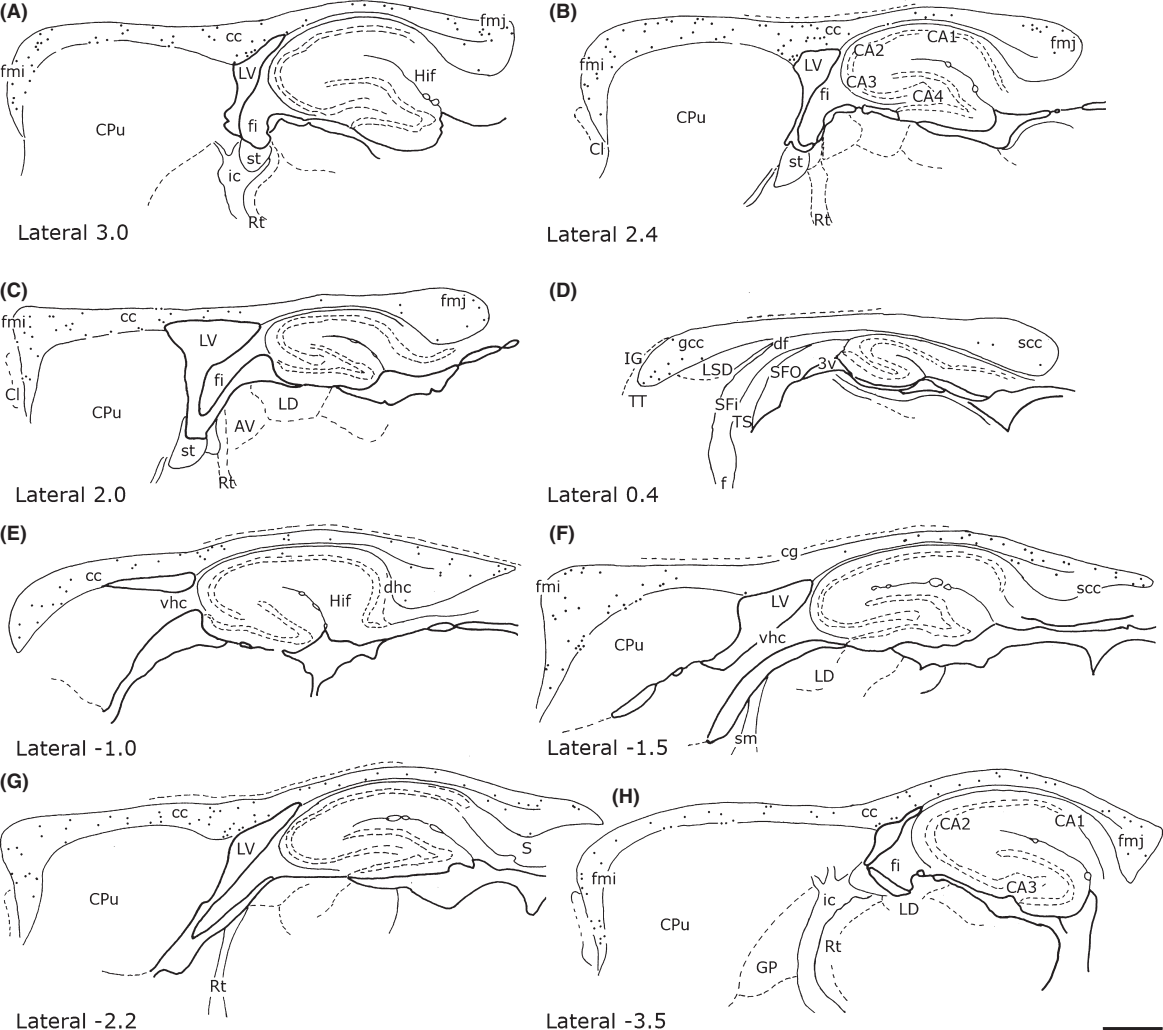
Distribution of NADPH-d+ neurons from lateral to medial (from A to D; right hemisphere) and from medial to lateral (from E to H; left hemisphere) in the rat corpus callosum. Stereotaxic coordinates according to the atlas of Paxinos and Watson (1982) on bottom left side. Calibration bar: 1 mm

**Figure 3:**
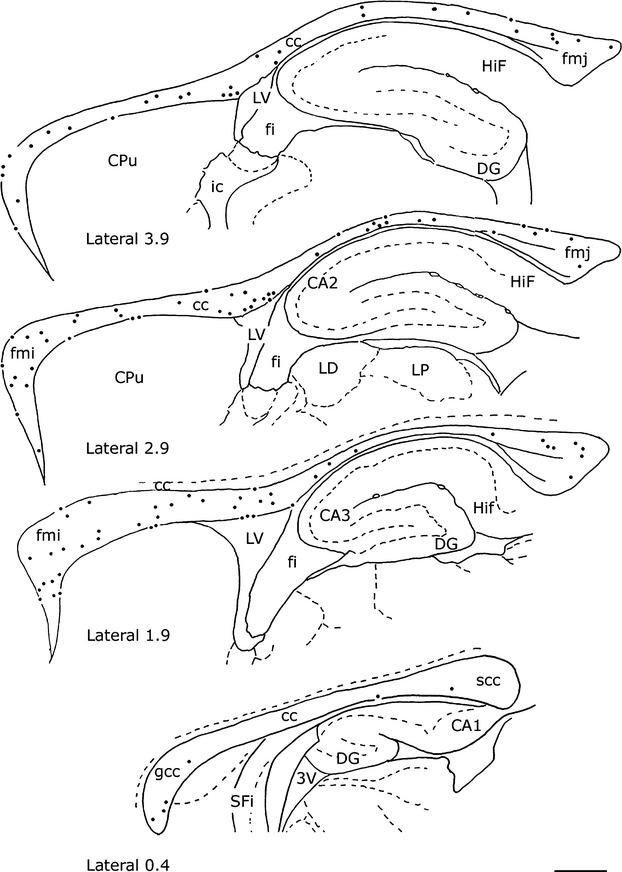
Distribution of nNOS-immunopositive (nNOS_IP_) neurons in the rat corpus callosum from lateral to medial. Stereotaxic coordinates according to the atlas of Paxinos and Watson (1982) on bottom left side. Calibration bar: 1 mm

**Figure 4:**
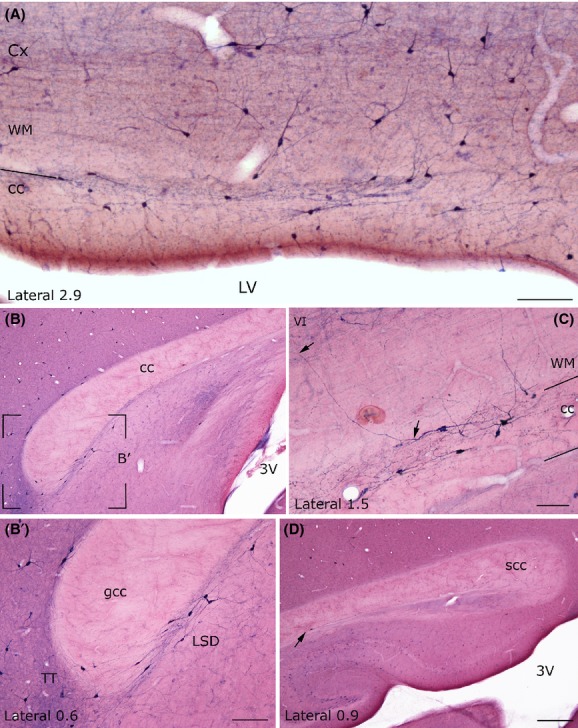
Low-power photomicrographs showing the distribution of NADPH-d+ neurons at different mediolateral levels of the rat corpus callosum. (A) Low-power photomicrograph showing many NADPH-d+ neurons in the lateral rat cc. (B) Absence of NADPH-d+ neurons at the medial level. The framed region in B is enlarged in B′. At this mediolateral level NADPH-d+ neurons are located around the genu. (C) Photomicrograph showing some positive neurons; one of them has a dendrite crossing the white matter and reaching layer VI. (D) Splenium of the rat cc showing a positive neuron (arrow). Stereotaxic coordinates according to the atlas of Paxinos and Watson (1982) on bottom left side. wm: white matter. VI, sixth layer of the cerebral cortex Calibration bars: 250 *μ*m in A, in B′ and C; 500 *μ*m in D.

NADPH-d+/NOS_IP_ neurons were isolated or formed groups with other NADPH-d+/NOS_IP_ neurons. In the splenium, they formed a cluster of 3–5 neurons, in the genu (or in the forceps minor of the cc), they formed a larger cluster located in the ventral cc on the boundary with the caudate-putamen or the lateral septal nucleus. NADPH-d+/NOS_IP_ dendrites branched in all directions, very often reaching the white matter and layer VI of the cerebral cortex (Fig. 4C and Fig. 6D) and/or the caudate-putamen nucleus or the hippocampus (see below), depending on the position of their perikaryon in the cc. Moreover, many NADPH-d+/NOS_IP_ located over the lateral ventricle sent dendrites as far as the ependymal layer.

**Figure 5:**
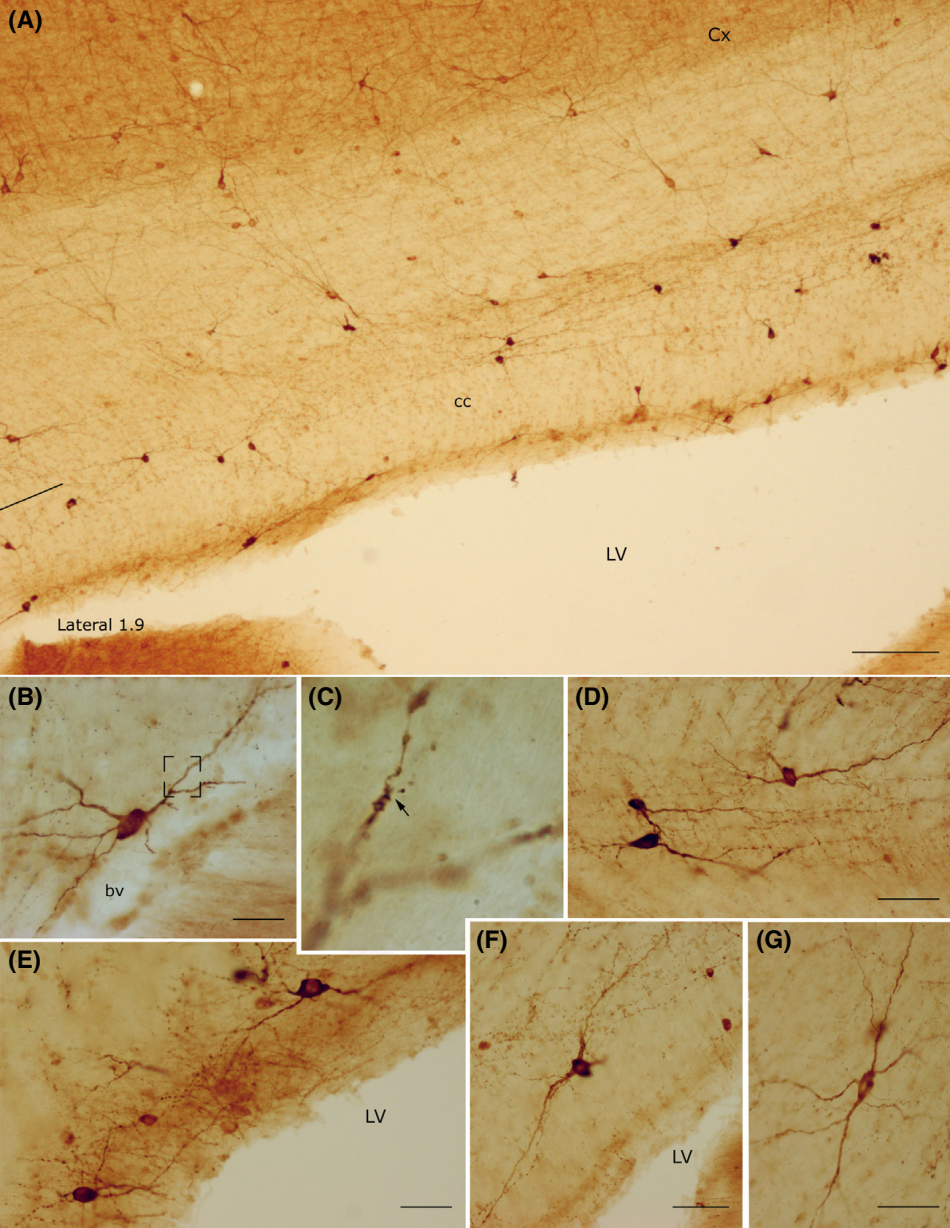
Photomicrographs of nNOS_IP_ neurons in the rat corpus callosum. (A) Low-power photomicrograph showing the distribution of nNOS_IP_ neurons. (B) A bipolar neuron close to an intracallosal blood vessel. Framed area enlarged in C. (C) Enlarged area showing a dendritic spine (arrowhead). (D) Triangular and ovoid nNOS_IP_ intracallosal neurons. (E) nNOS_IP_ neurons in the ependymal region. (F) A round nNOS_IP_ neuron near the lateral ventricle. (G) Bipolar nNOS_IP_ intracallosal neuron. Stereotaxic coordinates according to the atlas of Paxinos and Watson (1982) on bottom left side. Calibration bars: 250 *μ*m in A; 25 *μ*m in B–G.

**Figure 6:**
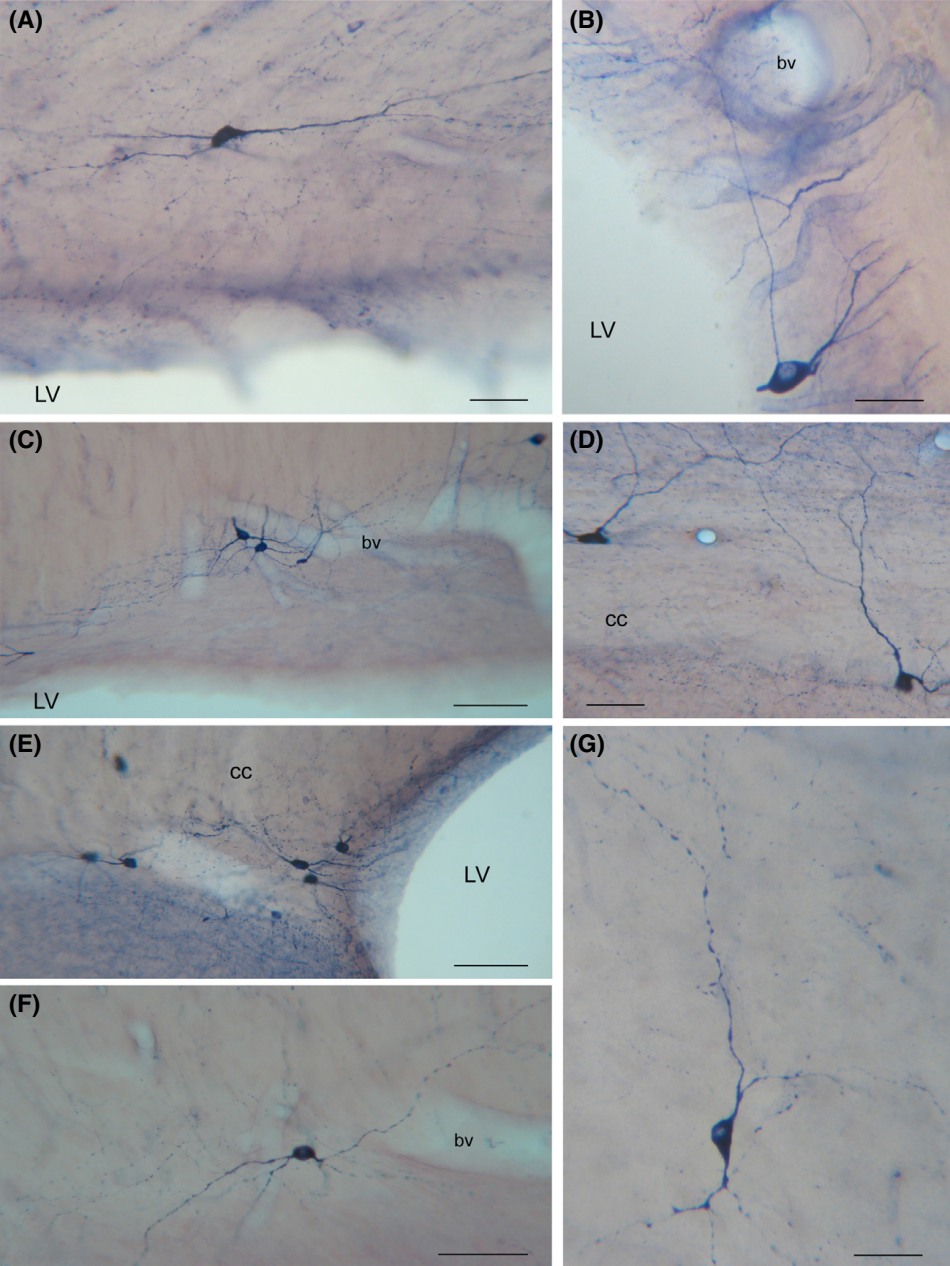
Morphology of NADPH-d+ neurons in the rat corpus callosum. (A) A bipolar NADPH-d+ intracallosal neuron with long dendrites extending along the rostrocaudal axis of the corpus callosum. (B) A pyriform NADPH-d+ neuron in the ependymal region. (C) Three NADPH-d+ neurons close to an intracallosal blood vessel. (D) NADPH-d+ neurons with vertically oriented dendrites. (E) A cluster of NADPH-d+ intracallosal neurons in the ependymal region. (F) A bipolar NADPH-d+ intracallosal neuron with dendrites close to a blood vessel. (G) An inverted pyriform NADPH-d+ intracallosal neuron with vertically oriented dendrites. bv, blood vessel. Calibration bars: 25 *μ*m for B and G; 50 *μ*m for A, D, F; 100 *μ*m for C, E.

Some NADPH-d+/NOS_IP_ neurons located in layer VI of the cerebral cortex, the white matter, or the caudate-putamen nucleus had dendrites reaching the cc. Bundles of labeled beaded processes that were not in continuity with neighboring NADPH-d+/NOS_IP_ neurons could be observed along the rostrocaudal extension of the cc. Several labeled neurons were also seen around the ependymal layer of the lateral ventricle (Fig. 5A and E, Fig. 6B and E).

Neurons positive for NADPH-d_Hi_ were counted in two brains (CC-NADPH-10, -11, both hemispheres; see Table 2), carefully avoiding including labeled neurons from the overlying white matter or the dorsal hippocampal commissure.

In CC-NADPH-10, neurons were counted in 145 50-*μ*m thick sections (accounting overall for 7250 *μ*m of thickness); in CC-NADPH-11, sections were 130 and their thickness was 50 *μ*m (overall 6500 *μ*m of thickness). In CC-NADPH-10, there were 2959 positive neurons (on average 20.4 neurons/section); of these, 2583 lay in the cc body and 376 (12.70%) in the ependymal region of the cc; in CC-NADPH-11, there were 2227 NADPH-d+ neurons (on average 17.1/section) of which 2029 were located in the body and 198 (8.89%) in the ependymal region. Ependymal neurons had a predominantly fusiform morphology. Counts performed in 278 pooled neurons from cases CC-NADPH-5, -7, -9 indicated that 46.76% (130/278) were fusiform, 25.17% (70/278) were polygonal, and that round and pyramidal neurons accounted for 19.06% (53/278) and 8.99% (25/278), respectively.

### Morphology of NADPH-d+ neurons

All NADPH-d+ neurons found in the cc were intensely stained and showed a Golgi-like appearance. Labeled neurons allowed studying the morphology of cc neurons whose somatic and dendritic characteristics enabled their classification into five distinct types: bipolar (fusiform, rectangular), round, polygonal (quadrangular), and pyramidal (triangular-pyriform).

### Bipolar neurons

These neurons were about 28.03% (see Table 3) of the entire population of NADPH-d+ intracallosal neurons.

Bipolar neurons can be subdivided into two categories:

- Fusiform, which accounted for about 24.44% (see Table 3).
- Rectangular, accounting for about 3.59% (see Table 3).

### Fusiform neurons

**Figure 7:**
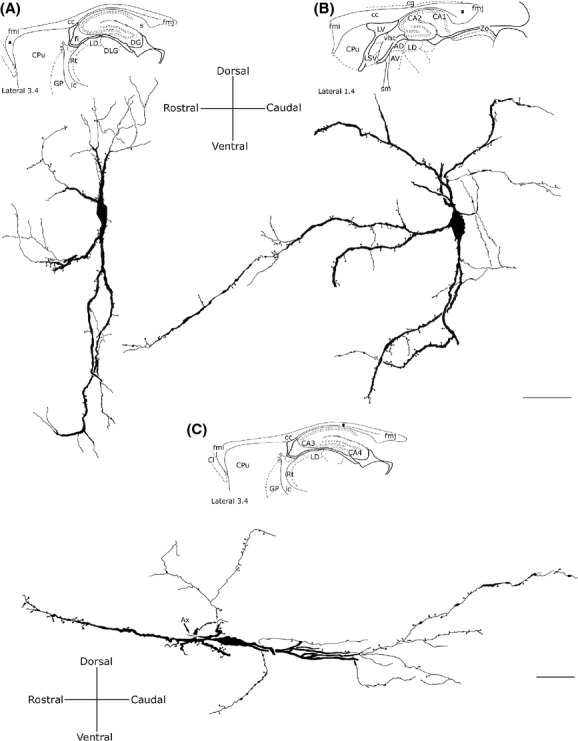
Camera lucida drawings of three bipolar (fusiform) NADPH-d+ neurons in the rat corpus callosum. Neurons in A and B are oriented vertically, neuron in C is oriented horizontally. Ax, axon. Calibration bars: 25 *μ*m.

Most perikarya of bipolar neurons found in the genu and splenium exhibited a similar size; their minor and major axis measured on average 10 *μ*m and 20 *μ*m, respectively. In these two cc regions, some fusiform neurons had a vertical orientation (Fig. 7A and B), while others were at a right angle to the former neurons (Fig. 7C). In the bipolar neurons found in the cc body, the minor axis was usually ∼5 *μ*m (Fig. 7C) and the major axis was oriented along the anteroposterior extension of the cc. From each pole of the cell body emerged one or two principal dendrites that gave off secondary and tertiary dendrites with abundant spines and fine dendritic processes on their surface (Fig. 7; for NOS_IP_ neurons see Fig. 5B and G). In some cases, primary dendrites emerged from the middle of the perikaryon (Fig. 6D, Fig. 7B; for NOS_IP_ neurons see Fig. 5B). Dendrites could be followed for several hundred microns: they bifurcated many times in progressively thinner smooth segments and often reached the white matter. When visible, axons originated from one of the two poles and could be followed only for tens of microns (Fig. 7C).

### Rectangular neurons

**Figure 8:**
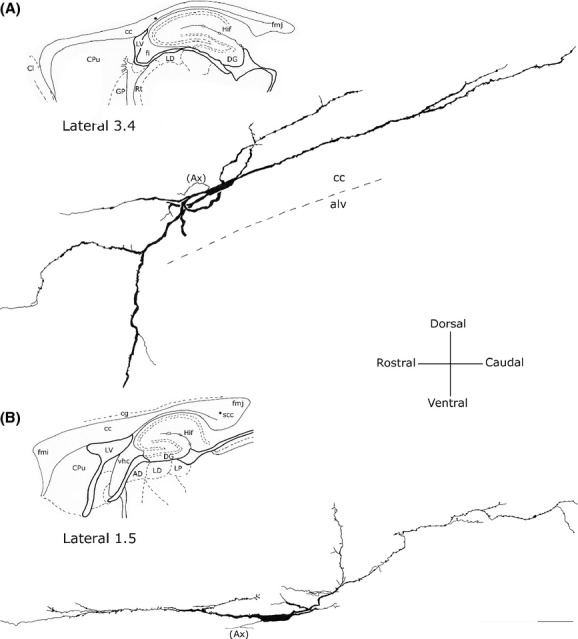
Camera lucida drawings of two rectangular NADPH-d+ neurons (A) in the middle and (B) splenium of the corpus callosum. A dendrite from the neuron in A reaches the alveus of hippocampus. Ax, axon. Calibration bar: 50 *μ*m.

These neurons had a long and narrow perikaryon, the longer side measuring 45–50 *μ*m and the shorter side about 10 *μ*m (Fig. 8). They were more frequently observed in the cc body or the splenium. One or two dendrites arose from the two poles of the soma and could be followed for several hundred microns in rostrocaudal direction. In some cases, secondary dendrites had a descending trajectory and crossed the inner portion of the cc to reach the alveus of the hippocampus (Fig. 8A); in other cases, they followed an ascending trajectory to the cortical white matter. Dendrites were smooth or carried a small number of spines. When visible, axons originated from one of the two poles and could be followed for no more than tens of microns (Fig. 8A and B).

### Round neurons

**Figure 9:**
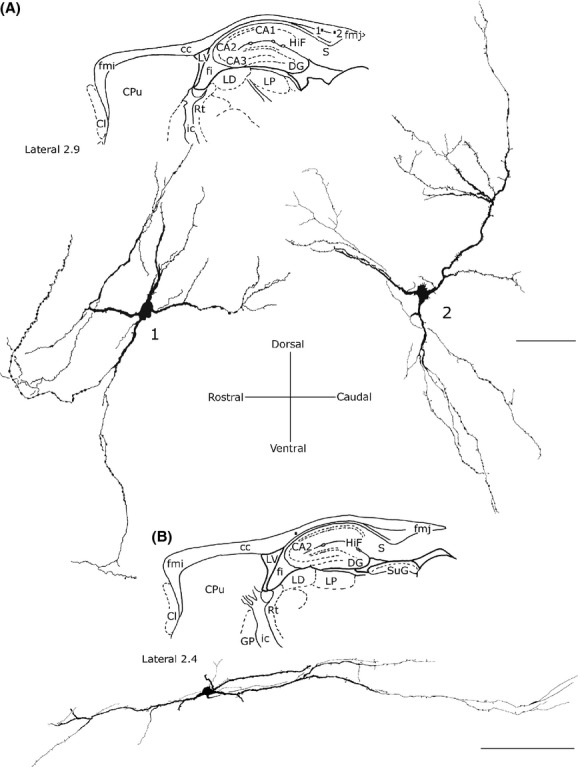
Camera lucida drawings of three NADPH-d+ round neurons. (A) Two of them (A-1 and A-2) lie in the forceps major of the corpus callosum; note the wide dendritic field. (B) Round neuron in the middle corpus callosum showing a narrow, elliptical dendritic field. Calibration bars: 50 *μ*m.

This morphological class accounted for about 19.26% of the entire intracallosal population labeled by NADPH-d histochemistry (see Table 3). These neurons had a round cell body, whose diameter ranged from 8 to 15 *μ*m, depending on their location in the cc (Fig. 6C, D, and F, Fig. 9A-2). Perikarya were usually smooth, but sometimes bore short appendages that were interpreted as somatic spines (Fig. 9A-2). Three to four thick dendritic trunks arising from the perikaryon of neurons located both in the genu and the splenium radiated outward in all directions, producing a wide, roughly circular dendritic field; dendrites arising from neurons located in the central third of the cc formed a narrow dendritic field oriented along the rostrocaudal extension of the cc. Secondary branches of the former neurons spread throughout the cc, reaching the overlying white matter. Proximal and distal branches were smooth or emitted rare spines; varicosities were observed in the distal regions of secondary and tertiary dendrites.

### Polygonal (quadrangular) neurons

**Figure 10:**
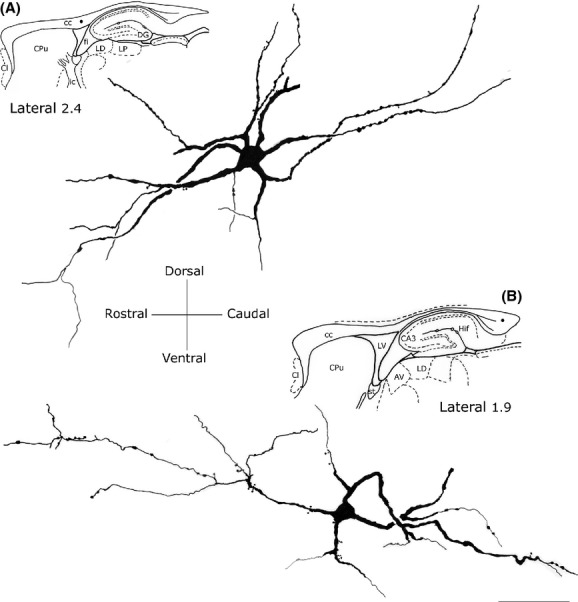
Camera lucida drawings of two polygonal NADPH-d+ neurons found (A) in the middle and (B) splenium of the corpus callosum wide dendritic fields taken from two different anteroposterior and mediolateral levels. Calibration bar: 50 *μ*m.

These neurons had a small (10–15 *μ*m) or large (20–25 *μ*m) polygonal or quadrangular soma (Fig. 10; for NOS_IP_ neurons see Fig. 5F). They were the most common cell type in the cc; counts performed in three cases (CC-NADPH-d-5; -7: -9), reported in Table 3, indicate that they accounted for about 30.11%. Thick dendrites emerged from the vertices of the soma and radiated in all directions to form a wide dendritic field. Dendrites (depending on the position of the soma) could be followed for several hundred microns as far as the cortical white matter, the caudate-putamen, the alveus of the hippocampus, or in many cases the ventricular surface. Dendrites were generally smooth, but some bore spines and fine dendritic processes (Fig. 10A and B). Occasionally, very thin axons were visible and could be followed for several tens of microns originating from the soma or, less frequently, from the base of proximal dendrites.

### Pyramidal (triangular-pyriform) neurons

**Figure 11:**
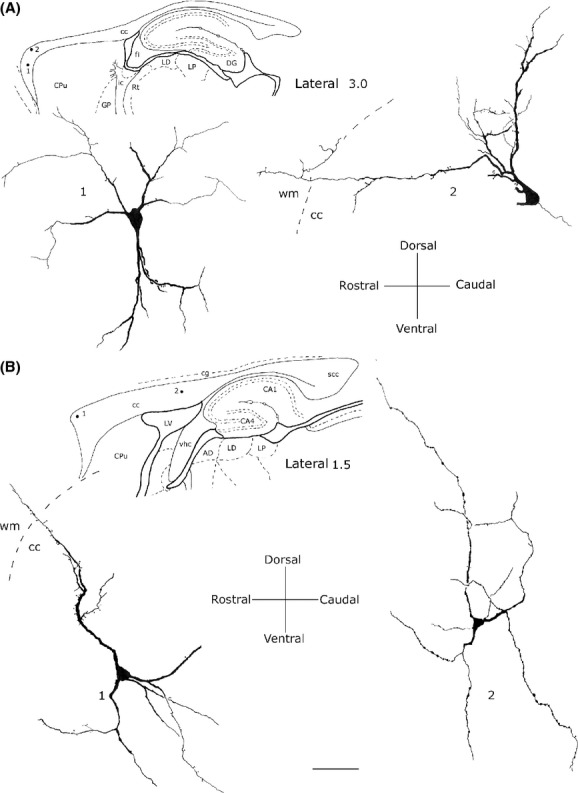
Camera lucida drawings of two pyriform (A1) and (A2) and two pyramidal (B1) and (B2) neurons from different callosal regions. Dendrites from neurons in A2 and B1 reach the overlying white matter (wm). Calibration bar: 50 *μ*m.

This class of neurons, accounting for about 22.58% of intracallosal neurons (see Table 3), had a triangular soma whose major axis ranged between 15 and 25 *μ*m. The soma of pyriform neurons (which were about 11.87%; see Table 3) had a drop-like perikaryon with an apical dendrite and one or two thick basal dendrites (Fig. 6G; Fig. 11; for NOS_IP_ neurons see Fig. 5D). In some instances, apical dendrites bifurcated after 25–30 *μ*m into two thick branches that traversed the cc to enter the overlying white matter (Fig. 11A-2). In other cases, the apical dendrite was directed toward the ventral region of the cc (inverted pyriform; Fig. 6G, Fig. 11A-1). Rare spines were observed in the dendritic arbor of these neurons. Thin axons, when labeled, originated from the base of the soma.

Triangular neurons were about 10.70% (see Table 3) and had a thick and wavy apical dendrite from which originated thin secondary spiny dendritic branches (Fig. 11B-1). Apical dendrites crossed the cc in the direction of the white matter, sometimes reaching it. The dendrites of some triangular neurons were completely devoid of spines.

The morphology of the intracallosal neurons whose dendrites reached the cerebral cortex could be studied in very few cases, as the extreme thinness of the distal dendrites and poor labeling made it difficult to follow their course. Overall 76 neurons were studied in cases CC-NADPH-5, -7, -9; of these 33 (43.42%) were polygonal, 17 (22.36%) were pyramidal, 15 were round (19.73%), and 11 were fusiform (14.47%).

### NO-producing neurons and blood vessels

**Figure 12:**
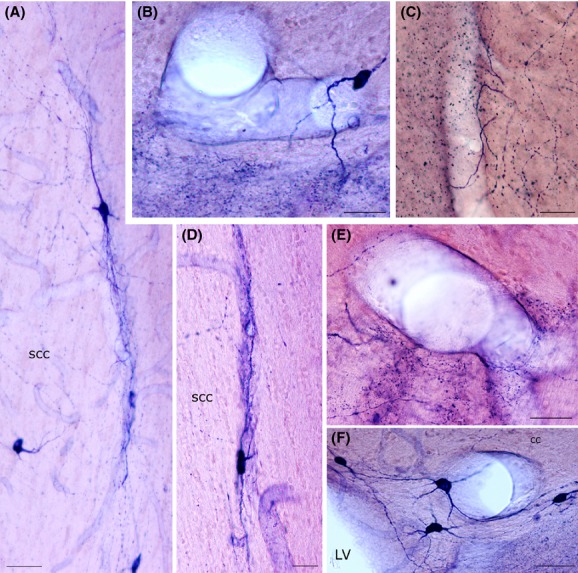
Photomicrographs showing NADPH-h+ neurons lying close to blood vessels. (A), (D) NADPH-d+ neurons in the splenium of the corpus callosum. Cell bodies and their processes are closely apposed to the wall of a longitudinal blood vessel. Photomicrographs are rotated 90° counterclockwise. (B) A NADPH-d+ neuron apposed to a callosal blood vessel encircles the wall with one of its processes. (C) A callosal vessel with stained fibers containing many varicosities and puncta. (E) Large blood vessel with stained fibers containing numerous varicosities and a spray-like distribution of NADPH-d+ puncta. (F) An intensely labeled callosal neuron wrapped around a blood vessel. Calibration bars: 25 *μ*m in A, B, C, D, E, and G; 50 *μ*m in F.

NADPH-d+/NOS_IP_ elements were closely associated with cc blood vessels (Fig. 6B, C, E, and F, Fig. 12). Counts performed in three NADPH-d experiments (NADPH-d-5; -7; -9; see Table 4) suggest that this population accounts for about 38% of all intracallosal NO-producing neurons. The most common morphological types close to blood vessels were polygonal (29.42%; see Table 4) and bipolar neurons (28.15%; see Table 4). Nerve fibers presenting varicosities along their course were detected in the adventitial layer of blood vessels, where they sometimes formed a tangled network (Fig. 12). Intensely labeled neurons were observed over or very near the vascular profiles, their cytoplasmic profiles wrapped around the walls of cc vessels (Fig. 12A, B, D, and F; for NOS_IP_ neurons see Fig. 5D). In other cases, intensely labeled cc neurons, at a distance from cc vessels, emitted long cytoplasmic fibers that could be followed as far as the neighboring blood vessel, where they formed a dense network (Fig. 6B; Fig. 12C and F). Often positive cytoplasmic processes found along intracallosal blood vessels were not attributable to any of the surrounding neurons.

### Double-labeling experiments (CC-Fl-1 and -2)

**Figure 13:**
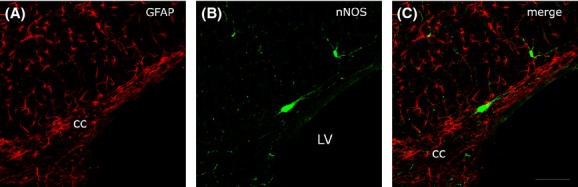
Confocal laser scanning photomicrographs showing the lack of colocalization (C) of GFAP+ (A, red fluorescence) and nNOS_IP_ neurons (B, green fluorescence) in the rat corpus callosum. Calibration bar: in C for A–C 25 *μ*m.

Colocalization of nNOS and GFAP was studied in two rats using confocal microscopy. Green fluorescent nNOS_IP_ neurons (Fig. 13B) showed morphological and distribution features consistent with those described in the preceding paragraphs. GFAP-immunopositive (GFAP_IP_) red fluorescent glial cells had their own typical morphology with a small soma and many long and thin processes; they were evenly distributed throughout the cc and were much more numerous than nNOS_IP_ neurons (Fig. 13A). nNOS neurons were scattered and intermingled with GFAP_IP_ cells; in either experimental case no cells contained both nNOS and GFAP (Fig. 13). These experiments indicated that: (1) NO-producing intracallosal cells are neurons; (2) glial cells do not contain nNOS.

## Discussion

The results of the present experiments can be summarized as follows:

- Double-labeling experiments disclosed that nNOS-positive cells did not contain GFAP, indicating that they may be considered as neurons.
- The rat cc contains numerous NO-producing neurons.
- NADPH-d+/nNOS_IP_ neurons show a lateromedial gradient, that is, they are more numerous in lateral than in medial cc regions.
- NADPH-d+/nNOS_IP_ neurons are morphologically heterogeneous.
- Many NADPH-d+ neurons are closely associated with intracallosal blood vessels.

The fluorescence experiments indicated that NO-producing cells in the rat cc are neurons. In both brains studied by this method, nNOS_IP_ neurons never contained GFAP and callosal glial cells did not contain nNOS. The presence of nNOS in glial cells in normal nervous tissue is debated. Previous studies have shown a very small proportion of nNOS_IP_ glial cells in the rat visual cortex (Lüth 1997) and guinea pig optic nerve (Qi and Guy 1996). Our study is in line with work performed in the rat intermediolateral cell column, where double-labeling experiments demonstrated the lack of nNOS/GFAP colocalization (Blottner and Baumgarten 1992).

### NO-producing neurons in the rat cc

The free radical NO is involved in many aspects of normal CNS functioning (Vincent 1994). NO is synthesized from l-arginine by three different isoforms of the NOS enzyme: nNOS, endothelial NOS (eNOS) and inducible NOS (iNOS) (Garthwaite 1991; Moncada et al. 1991; Knott and Bossy-Wetzel 2009). Neuronal NOS is found in CNS and peripheral nervous system neurons; NO-producing neurons can therefore be visualized by immunocytochemical nNOS detection. Moreover, nNOS activity can be evaluated using a histochemical stain for the enzyme NADPH-d, which is considered a neuronal NOS (Hope et al. 1991). We therefore used immunocytochemistry and histochemistry to investigate the presence, distribution, and morphology of NO-producing neurons in the rat cc. Our findings partially confirm those obtained in monkey (Rockland and Nayyar 2012), but in addition, they provide a detailed description of the number and distribution of NO-producing neurons.

Special attention was devoted to defining cc boundaries, especially in the most lateral stereotaxic planes, where it is harder to discriminate the cc dorsal border from overlying white matter. To achieve a better visualization of this border, sections adjacent to those reacted for NADPH-d/NOS were processed for CO and counterstained with neutral red. Axon trunks, in general, whether myelinated or unmyelinated, have relatively low levels of CO activity (Wong-Riley 1989) and seemed to have lower CO activity in the cc than in the overlying white matter. Borders defined by CO activity were collated with those from sections counterstained with neutral red. Reconstructions of the cc were then compared with those reported in the stereotaxic atlas of Paxinos and Watson (1982). These criteria were applied to study the distribution and number of intracallosal neurons.

Neurons were detected throughout the rostrocaudal dimension of the cc, but showed a mediolateral gradient, being more numerous in the lateral region and rare in the medial region. No comparative data are available on differences in regional distribution of NADPH-d/NOS intracallosal neurons between rodents and primates, because Rockland and Nayyar (2012) limited their study to the medial-most region of the monkey cc. However, the present findings and those of a previous study suggest that the adult rat cc contains different neuronal populations which could all have a distinctive areal distribution; indeed MAP2+ neurons are mainly concentrated in the rostrum near the border with the septum pellucidum (Riederer et al. 2004).

Counts performed in two brains (both hemispheres) indicated that the rat cc contains a substantial population of NADPH-d+ neurons. Counts involved 145 sections in brain CC-NADPH-10 and 130 in brain CC-NADPH-11, yielding 2959 NADPH-d+ neurons in the former and 2227 NADPH-d+ neurons in the latter. Previous immunocytochemical studies documented numerous intracallosal neurons in the developing cc of various species, including humans, that decline in the postnatal period (DeDiego et al. 1994; Deng and Elberger 2001; Misaki et al. 2004; Riederer et al. 2004; Niquille et al. 2009; Jovanov-Milosevic et al. 2010). However, intracallosal NO-producing neurons likely have a different postnatal behavior, like those found in the visual cortex white matter, where number and density of nNOS_IP_ cell increase during postnatal development (Clancy et al. 2001).

We found 8–12% NO-producing neurons in the ependyma of the lateral ventricle. These neurons may be in contact with CSF through their dendrites, axons or perikarya, and may belong to the CSF-contacting neuronal system found in various periventricular brain regions of vertebrates (Vigh et al. 2004). Ependymal neurons were predominantly fusiform. This finding agrees with a previous NADPH-d study (Sancesario et al. 1996) They could have a sensory function, registering CSF composition; play a negative feedback role on CSF pH and osmolality changes; or else be involved in regulating the synthesis and release of several peptides in the CSF (Westergaard 1972; Sancesario et al. 1996; Xiao et al. 2005).

NADPH-d+ neurons had a darkly stained cell body and dendrites that lent them a Golgi-like appearance. Based on previous species-specific studies, the intracallosal population could be classified as type 1, defined by a dense NADPH-d histochemical reaction (Yan et al. 1996); no type 2 neurons, characterized by low-level NADPH-d activity (Yan et al. 1996), were observed in the cc. Their dendritic trees and the morphology of the perikaryon enabled classification of NADPH-d+/NOS_IP_ neurons into five groups: bipolar (fusiform, rectangular), round, polygonal (quadrangular), and pyramidal (triangular-pyriform). Our data therefore indicate that in the rat cc, as in the monkey (Rockland and Nayyar 2012), there exists a wide neuronal heterogeneity that is actually based only on morphological criteria. The heterogeneity of NO-producing neurons in the cerebral cortex is based on different criteria. NADPH-d+/NOS_IP_ neurons belong to one of two classes, type 1 or type 2 (Yan et al. 1996), based on their content in NO-producing enzymes. Moreover, nNOS–type 1 neurons display fast-spiking activity, they account for 0.5–2% of the cortical GABAergic population, and in these neurons nNOS is associated with somatostatin and neuropeptide Y (for a review see Tricoire and Vitalis 2012). It cannot therefore be excluded that the NADPH-d/nNOS-type 1 neurons found in the rat cc are characterized by chemical heterogeneity. Further double-labeling studies are in progress in our laboratory to test this hypothesis. However, chemical heterogeneity has been observed in cc neurons, especially in the early stages of postnatal life; some intracallosal neurons contain calretinin, calbindin, GABA, and MAP2 (DeDiego et al. 1994; Riederer et al. 2004).

Intracallosal neurons have a wide dendritic field with many dendrites extending into white matter. In the best cases, they could be followed up to layer VI of the overlying cerebral cortex; they may thus receive synaptic inputs from different sources. Collaterals of cortical afferent and efferent systems could terminate on these dendrites, a hypothesis that is supported by previous studies. An anterograde tracer injected into different cortical areas anterogradely labeled synaptic terminals establishing synapses on white matter interstitial neurons (Clarke et al. 1993; Shering and Lowenstein 1994). Moreover, both thalamocortical and claustrocortical afferents, which form a dense plexus in layer VI (Zhang and Deschênes 1998; Arnold et al. 2001; Oda et al. 2004), could contact the dendrites of intracallosal neurons, which could thus receive a synaptic input also from neurons located in layer VI—whose axon is confined to the same layer—and/or from collaterals of corticothalamic axons (Briggs 2010). As intracallosal neurons are fully embedded in callosal fibers, another source of influence could be the callosal fibers themselves. Recent evidence suggests that callosal fibers are able to release vesicular glutamate at discrete sites along their course, which could affect NO-producing neurons as well as glial cells (Kukley et al. 2007; Ziskin et al. 2007). This hypothesis is in line with previous findings suggesting that nNOS produces NO after stimulation of NMDA glutamate receptors (Garthwaite 1991; Vincent 2010).

Although in many cases the axon of intracallosal neurons could be followed only for some tens of microns, previous studies combining retrograde labeling and immunocytochemistry indicate that NADPH-d+/nNOS_IP_ neurons have axons extending for thousands of microns that are part of the corticocortical network (Tomioka et al. 2005; Tomioka and Rockland 2007). Therefore, intracallosal neuron axons could be confined to the cc—connecting other intracallosal neurons that lie far apart and forming an integrated network that could influence the flow of neuronal impulses along callosal fibers—or they could reach the cerebral cortex. These cells form a substantial population which amounts to 38% of the intracallosal population neurons.

One of the most interesting features of NADPH-d+/nNOS_IP_ neurons is their close association with blood vessels. These cells form a substantial subpopulation, accounting for about 38% of the entire NADPH-d+ callosal population. However, as in many cases it was impossible to relate the NADPH-d+ cytoplasmic processes to any labeled cell body, the proportion may be underestimated.

The soma of NADPH-d+/NOS_IP_ intracallosal neurons was seen to be apposed to callosal vessels and their axonal plexuses formed a dense network around vessels. The close association of NADPH-d+/NOS_IP_ elements with callosal vessels is in line with the physiological area of NO influence, which is ∼100–200 *μ*m (Wood and Garthwaite 1994; Estrada and DeFelipe 1998). As NO is a potent vasodilator, nNOS-containing neurons are thought to be involved in coupling metabolic changes related to neuronal function with local increases in blood flow (Iadecola 2004).

The neurovascular interactions inducing hemodynamic changes during variations in cortical activity underpin functional neuroimaging with positron-emission tomography (PET) and functional magnetic resonance imaging (fMRI; Suárez-Solá et al. 2009; Iadecola 2002, 2004). The blood oxygen level-dependent (BOLD) signal reflects the hemodynamic responses coupled to neuronal signaling processes (Iadecola 2004; Lauritzen 2005).

The exact mechanism underlying the BOLD effect is still debated. It may be hypothesized that hemodynamic changes induced by motor and visuomotor tasks and peripheral stimulation (Mosier and Bereznaya 2001; Tettamanti et al. 2002; Omura et al. 2004; Weber et al. 2005; D'Arcy et al. 2006; Mazerolle et al. 2010; Fabri et al. 2011) in specific cc regions could be related to the presence of NADPH-d+/NOS_IP_ intracallosal neurons, whose depolarization could cause an increase in blood flow.

Such depolarization could occur in two different ways: (1) excitation of some cortical regions due to peripheral stimulation could induce depolarization of those NADPH-d+/NOS_IP_ intracallosal neurons whose dendritic trees reach the activated overlying cerebral cortex; this in turn could induce NO release from neuronal processes associated with intraparenchymal callosal vessels, as proposed for the cerebral cortex, where nNOS inhibition attenuates the increase in blood flow associated with neuronal activity (Iadecola et al. 1993; Estrada and DeFelipe 1998; Attwell et al. 2010); (2) during an increase in cortical activity callosal fibers, consistent with their origin from glutamatergic neurons (Barbaresi et al. 1987), release glutamate along their course (Kukley et al. 2007; Ziskin et al. 2007) possibly excitating NO-producing intracallosal neurons (Iadecola and Nedergaard 2007) through NMDA receptors (Garthwaite 1991, 2008); interaction of glutamate with NMDA receptors could therefore be necessary for BOLD responses in the cc as in other CNS regions, where application of NMDA receptor antagonists attenuates blood flow responses (Iadecola et al. 1996; Nielsen and Lauritzen 2001; Gsell et al. 2006; Hoffmeyer et al. 2007; Busija et al. 2007; Tiede et al. 2012).

However, a concomitant role of astrocytes in neurovascular coupling (Attwell et al. 2010) in the cc cannot be ruled out. As the present findings indicate that glial cells lack NO-producing enzymes, glutamate released from callosal axons could induce release of vasoactive agents other than NO from astrocyte end feet, like for example, cyclo-oxygenase (COX) products, whose inhibition significantly reduces vasodilation (Zonta et al. 2003; Takano et al. 2006).

## Conclusion

In summary, we demonstrated that the adult rat cc contains a sizeable population of NADPH-d+/nNOS_IP_ neurons amounting to over 2000 intracallosal cells. Their distribution shows a lateromedial gradient, a greater number of neurons being found in the lateral stereotaxic planes than in the more medial ones. NADPH-d+/nNOS+ intracallosal cells present a considerable morphological heterogeneity. In addition, their location in the ependymal regions of the cc and their association with intracallosal blood vessels suggests an active role for them in regulating both CSF composition and intracallosal blood vessels.
